# Lithotomy in the Female. The Various Operations, &c.

**Published:** 1864-09

**Authors:** 


### CHRONICLE OF MEDICAL SCIENCE.

Art. I.—*Lithotomy in the Female. The Various Operations, &c.*

Various operations have been proposed and practised in this disease; but many of them having proved objectionable and dangerous have fallen into disuse. The supra-pubic or high operation, being most dangerous of all, is almost entirely dismissed from the practice of surgery. The only conceivable case in which it would be justifiable would be, perhaps, one in which, with a deformed pelvis and a very large stone, contraction per vaginum was impracticable.

*Dilatation of the uretha* has of late years been more resorted to than any other. Its facility renders it very tempting to the surgeon, while its safety and bloodlessnes recommends it strongly to the patient. The consequence is that it has caused many an unfortunate woman to pass the rest of her days in a loathsome and miserable condition. The female urethra is easily dilatable, though perhaps beyond a limited point the word *lacerable* might express what occurs more correctly. Small, and even large stones have frequently been extracted by this method without permanent injury to the functions of the urethral carnal. But it is nevertheless admitted on all hands that the urethral method is frequently followed by inability to retain the urine. The dilatation paralyzes, or in some way injures, the sphincter fibres at the neck of the bladder, and this produces an incontinence, which, though it may sometimes be spontaneously recovered from, not unfrequently persits for the remainder of the patient's life. She is freed from a painful disease, but left in a condition which renders life scarcely worth having, and which science is powerless to relieve. It is impossible to say beforehand how much distension any particular uretha will endure without permanent and irreparable injury. Urethral dilatation should, therefore, be only practiced in cases where the stone is of small size.

*Incision of the urethra* is the next method to be considered, and any incision of this carnal in a *downward* direction, which would lay any portion of it open into the vagina is wrong in principle and pernicious in practice. It gives no additional room for the extraction of a stone, and is therefore useless; while its thin divided edges are certain not to unite spontaneously, and it is exceedingly difficult to procure their union by a plastic operation. But though incision of the urethra *downwards* towards the vagina is in every way objectionable, it is otherwise as regards incisions made in an *upward* direction. By dividing the urethra and neck of the bladder towards the symphisis pubis, room may be obtained for the extraction of a moderate sized stone, without injury to any important structure, and without the disadvantage of laying one mucous channel open into another, when the two are only separated naturally by a very thin partition. Brodie, Crampton and Liston combined incisions upwards with dilatation. But if any incision of the urethra is to be made, it is better to depend upon incision alone, rather than combine it with dilatation, as by so doing the chance of incontinence would be diminished. Of all the urethral methods, the incision upwards is the only one at all deserving of confidence. It is, perhaps, not altogether devoid of the risk of urinary infiltration into the loose areolar tissue between the bladder and the symphisis pubis; but the urethra, which is situated at the bottom of the incision, forms so convenient a groove for the escape of urine that this risk is probably not very great.

*The Lateral Operation.*—A method which, though it cannot be called new, is not at all generally known, and not so well known as it deserves to be. It is, as nearly as the different structure of the parts will permit, the exact counterpart of the lateral operation in the male. It may be performed in the following manner: A straight grooved staff having been introduced into the bladder, an incision is to be made on the inner surface of the left nympha, commencing half an inch above the meatus urinarius, and passing obliquely downwards and outwards parallel with the rami of the pubes and ischium. This incision should be carried deeply into the space between the rami on the outside and the vagina on the inside, care being taken not to wound the vagina, which should be protected and pushed inwards towards the median line by the left forefinger introduced into the wound. With the same finger the staff should be felt for deep in the wound, and the knife should be made to penetrate the groove at a point corresponding, as nearly as can be judged, to the vesical termination of the urethra. It should then be passed onwards into the bladder, its cutting edge being turned obliquely downwards and outwards towards the left side, just as in lateral lithotomy in the male. The incision may be enlarged should it be thought necessary, as the knife is withdrawn, by a further division of the tissues in the same oblique direction. The ordinary lithotomy forceps can then be introduced into the wound for the extraction of the stone.

This operation, or something like it, seems to have been practiced by Frere Jacques. It has been condemned in succession by several writers of notice, but evidently upon insufficient grounds. Its revival, and it may be almost said, its introduction, is due to Dr. Andrew Buchanan, of Glasgow, who, it appears, has operated in this way in six cases, with very satisfactory results, both as regards the healing of the wound and the subsequent ability to retain the urine. The risk attending it is, as nearly as possible, the same as lateral lithotomy in the male, which in children we know is not very alarming. It is not likely to cause any permanent injury to the retentive power of the bladder, as no part of the bladder or urethra is laid open into the vagina; while the track of the wound, passing, as in the male, through a considerable depth of tissue, is pretty certain to heal by granulation.

The proceeding which has been described as the *vestibular operation*, which is said to have been practiced by Celsus, and which revived Lisfranc, possesses sufficient interest as one of the modes by which the bladder may be reached as not to be passed over in silence; but it is objectionable in many respects, and possessing no independent advantages, it has been very properly laid aside. The vestibula is the triangular smooth space bounded above by the clitoris, below by the meatus urinarius and the commencement of the vagina, and on each side by the nympha. An ordinary grooved staff is introduced into the urethra, with its convexity turned upwards towards the symphisis pubis—that is to say, its ordinary position, as used in the male, is reversed. A curved incision, with its convexity upwards, or parallel with the arch of the pubus, is then to be made across the centre of the vestibular space, and the tissues intervening between the pubes above and the urethra below to be divided, until the grooved staff can be felt in the urethra near its vesical termination. The urethra is to be opened near the neck of the bladder, on its upper surface, by cutting into the groove of the staff, and the incision is to be extended through the neck of the bladder, either in the transverse or upward direction.

The obvious disadvantages of this method are, that the incision has to be made through so inconveniently narrow a space, and that the stone has to be extracted through the narrowest part of the arch of the pubes. The confined condition of the wound must also cause considerable risk of urinary infiltration about the neck of the bladder. The plan, indeed, appears to have been ultimately abandoned by Lisfranc himself.

The operation which promises the best results, when applicable, is the *vesico-vaginal*, which is described by Mr. Lane in the following manner: The patient being placed in the lithotomy position, Boveman's speculum is introduced into the vagina, and held so as to expose its anterior wall, and a straight grooved staff is passed into the bladder by the urethra. An incision is then made into the groove of the staff, commencing just behind the neck of the bladder, and extending backwards in the median line for about an inch and three-quarters—to within a short distance, in fact, of the attachment of the vagina to the cervix uteri. Through such an incision, a stone two inches in length may be readily extracted. The edges of the wound are then brought into opposition by silver wires. No object is attained by commencing the incision at the external orifice of the urethra; indeed it cannot be too strongly urged that this canal on no account be interfered with. The incision should commence just behind the neck of the bladder; that is to say, in an adult person at least an inch and a quarter behind the external orifice of the urethra. Incision of the urethra gives no additional room, but increases the length of the wound without any compensating advantage whatever, and the urethral portion of the wound is incomparably more difficult to close than that which is deeper in the vagina.

The main objections to this operation were formerly the risk of its being followed vesico-vaginal fistula. This was Velpeau's objection. Now, however, all this is changed. The experience of the last ten years has abundantly shown that almost every case of vesico vaginal fistula, even when attended with great loss of substance, may be firmly and permanently closed by the improved plastic procedures now in use. We may, therefore, very fairly feel confidence in our ability to close the clean longitudinal incision made for the extraction of a stone, which is attended by no loss of substance, and with no cicatrical contraction or induration.

The conclusions which may be formed with regard to this operation are, that in an adult female, and especially in the case of a large stone, lithotomy through the vagina, conducted on proper principles, and followed by immediate closure of the wound, is the safest and best procedure that has as yet been devised, and deserves to be accepted as a recognized operation in surgery. It should be understood however, that it is not applicable to children; neither is it well adapted for young unmarried women, in whom the difficulties of this operation must necessarily be greatly increased.

We will terminate these remarks by submitting for consideration the conclusions which have been formed with reference to the subject of lithotomy in the female. bearing in mind always that, though the first object is undoubtedly to remove the stone with as little risk as possible to the life of the patient, yet that it is almost of equal importance that this should be done without permanent injury to the retentive function of the bladder and urethra. These conclusions are briefly as follows:

1st. That dilatation of the urethra should only be employed for the removal of stones of very limited size; otherwise it is attended with serious risk of incontinence of urine, *that incontinence being incurable.*

2d. That incisions of the urethra in the *downward* direction should be discarded altogether. But that the incision *upwards* may be practised with little danger to life, and little risk of incontinence of urine It is not, however adapted for the removal of stones of any considerable magnitude, neither is it well suited for children. Free incision are preferable to any combination of incision with dilatation.

3d. That the vesico-vaginal incision, with immediate closure of the wound by suture, is admirably adapted for the removal of stones in the case of adult women, in whom the vagina is of average capacity; that it is the only safe and available method for the removal of stones of large size; that it is attended with a minimum of immediate risk, and no risk at all of *permanent* incontinence of urine.

4th. That the lateral operation of Dr. Buchanan is founded upon sound anatomical and surgical principles, and is probably the best operation that can be practiced in children. It is also well adapted for young and unmarried women, in whom the small size of the vagina would contra-indicate the vesico-vaginal method. In these latter cases the choice would therefore lie between the lateral operation and the incision upwards of the uretha.

5th. That there are scarcely any conceivable circumstances which would render the high operation above the pubes justifiable.

6th. That the "vestibular" operation of Lisfranc possesses no merit of its own to compensate for its manifest disadvantages.

